# Low tumoral Trefoil Factor 1 expression relates to aggressive tumor features and poor survival in young women with breast cancer

**DOI:** 10.1038/s41598-026-36341-4

**Published:** 2026-01-17

**Authors:** Amalie B. Kvamme, Ulrikke Hugaas, Anna K. M. Sæle, Lise M. Ingebriktsen, Amalie A. Svanøe, Rasmus O. C. Humlevik, Ingeborg Winge, Turid Aas, Anette Heie, Lars A. Akslen, Erling A. Hoivik, Elisabeth Wik

**Affiliations:** 1 Centre for Cancer Biomarkers CCBIO, Department of Clinical Medicine, Section for Pathology, University of Bergen, N-5021 Bergen, Norway; 2 Department of Pathology, Haukeland University Hospital, Bergen, Norway; 3 Department of Medical Biochemistry and Microbiology (IMBIM), Uppsala University, Uppsala, Sweden; 4 Department of Surgery, Haukeland University Hospital, Bergen, Norway; 5 Department of Oncology and Pathology, Karolinska Institutet, Stockholm, Sweden

**Keywords:** Breast cancer, TFF1, Young age, Survival, Biomarkers, Cancer, Oncology

## Abstract

In young breast cancer (BC) patients, there is a need for additional biomarkers beyond those currently available. Trefoil Factor 1 (TFF1) is an estrogen-regulated protein with proposed prognostic relevance in BC. We assessed TFF1 protein expression by immunohistochemistry (IHC) in biopsies from patients with primary breast cancer diagnosed before age 50 and their paired axillary lymph node (LN) metastases within our in-house Bergen Young BC Cohort (n = 319). In addition, TFF1 mRNA levels and associated gene expression patterns were analyzed using publicly available datasets, including the Molecular Taxonomy of Breast Cancer International Consortium (METABRIC, n = 939) and the Cancer Cell Line Encyclopedia (n = 47). Our aim was to investigate how TFF1 expression in primary tumors (PT) and paired LN metastases relates to age at diagnosis, clinico-pathological features, and disease-specific survival, and to explore TFF1-associated gene expression profiles. Low TFF1 protein and mRNA expression in PTs was associated with younger age, aggressive clinico-pathologic features, and poorer survival. Additionally, TFF1 mRNA expression was a strong predictor of the basal-like subtype, and TFF1-low tumors exhibited stem-like characteristics, upregulation of immune checkpoints, and higher proliferation scores. In LN metastases, low TFF1 protein expression was associated with aggressive tumor features. Notably, high TFF1 protein expression in LN metastases predicted poorer survival in patients under 40 years. Our results indicate that low BC TFF1 expression is associated with younger age and aggressive tumor features, on top of serving as a predictor of the basal-like subtype. High TFF1 protein expression in LN metastases may carry prognostic significance for younger patients.

**Supplementary Information:**

The online version contains supplementary material available at 10.1038/s41598-026-36341-4.

## Introduction

Breast cancer (BC) is a large contributor to global health burden among females and one of the leading causes of death among women aged 25–49^1,2^. Younger patients face worse prognosis, more aggressive disease, poorer response to treatment, and higher risk of recurrence compared to BC patients above 50 years of age^3^. Despite this, the underpinning differences in tumor biology between younger and older women are still poorly understood, and there is a lack of robust markers of aggressive disease and clinical outcome tailored for this patient group^4^. Current knowledge about treatment and prognosis is largely based on research conducted on older BC patients, without specifically considering the young^5^.

The expression of Estrogen receptor α (ER, encoded by *ESR1*) and Progesterone receptor (PR, encoded by *PGR*), Human Epidermal Growth Factor Receptor 2 (HER2; encoded by *ERBB2*), and the proliferation marker Ki67, are routinely assessed for prognostic and predictive purposes^6,7^. Notably, ER-related proteins like GATA binding protein 3 (GATA3), Anterior Gradient 2 (AGR2) and TFF1^8–13^, have to some extent been studied in BC of the young. However, much is still unknown about ER-related expression and signaling, including how such markers relate to age and biology of young BC patients^4,14^.

Trefoil Factor 1 (TFF1) is a secretory protein found in normal gastric mucosa where it is thought to exert anti-inflammatory and anti-tumorigenic functions^15,16^. TFF1 occurs in several types of malignancies, including BC^17–20^. Transcription of TFF1 is directly regulated by estrogen through ER binding at its promoter^13,21^, and its expression is associated with ER positivity and luminal BC subtypes^17,18,20^. However, the role of TFF1 in estrogen signaling and BC is still uncertain^8,22,23^. Some reports suggest TFF1 to be a marker for metastasis^24–27^, while others suggest TFF1 to inhibit migration and metastasis^28^. However, even though there are many studies on TFF1, few studies have investigated the expression specifically in a younger population. In our study, we aimed to better understand TFF1 expression in BC of younger patients by examining TFF1 protein by immunohistochemistry (ICH) in biopsies from primary tumors (PT) and lymph node (LN) metastases in our in-house Bergen Young BC Cohort (n = 319), exploring the biological processes associated with TFF1 by mRNA and gene expression analyses on publicly available datasets The Molecular Taxonomy of Breast Cancer International Consortium (METABRIC, n = 939) and Cancer Cell Line Encyclopedia (CCLE, n = 47), and evaluating its value as a prognostic biomarker in this patient population.

## Results

### Low TFF1 protein and mRNA expression is associated with young age, aggressive tumor features and poor survival outcome

**Fig. 1:**
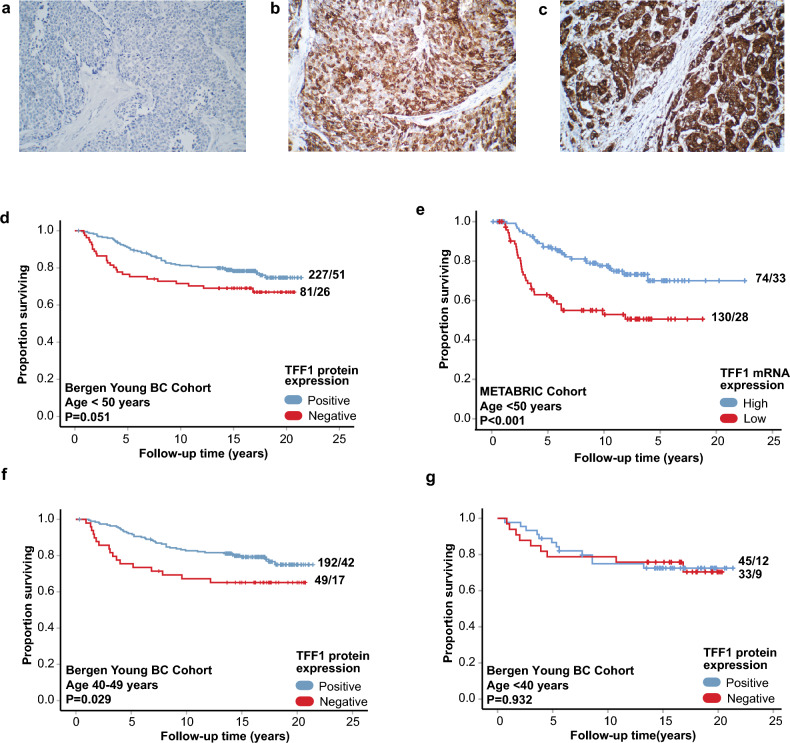
TFF1 in relation to immunohistochemical staining and survival. (**a–c**) TFF1 IHC protein as negative, heterogenous/moderate and strong staining intensities, respectively, in infiltrating ductal carcinoma. (**d–e**) Association between TFF1 and survival in the Bergen Young BC Cohort and the METABRIC cohort. (f-g). Association between TFF1 and survival in the Bergen Young BC Cohort in patients aged 40–49 and < 40 years.

TFF1 protein expression was determined by IHC and found to be expressed in the cytoplasm of cells (in-house Bergen Young BC Cohort). A heterogenous intratumor expression pattern was observed in both invasive carcinoma and in situ components (Fig. 1a–c), as well as in normal breast epithelium. TFF1 expression was evaluated using a semi-quantitative scoring system—the staining index (SI)—based on the combined evaluation product of staining intensity (scored 0–3: no staining, weak, moderate or strong) and the fraction of positive tumor cells (0–3: < 10%, 10–50%, or > 50%) into a total score of 0–9^29,30^. Tumors with no detectable TFF1 expression (SI = 0, denoted TFF1 negative) accounted for 25.7% of the cases.

Negative TFF1 protein expression was associated with age below 40 years (OR 2.87, P < 0.001, Table 1). Among patients below 40 years, 42.3% (33/78) presented negative TFF1 expression in the PT, compared to 20.3% (49/241) of patients aged 40–49 years (Table 1). Furthermore, negative TFF1 was associated with high histologic grade, ER and PR negativity, and the HER2 and triple negative subtypes (OR ≥ 1.13, all P < 0.001, Table 1).

**Table 1:** Association between TFF1 protein expression (IHC) and selected characteristics in breast cancer (n = 319; Bergen Young BC Cohort). n: number of patients; LN: lymph node; OR: odds ratio; CI: confidence interval: p-values by Pearson’s chi-squared test. Missing information: tumor diameter: n = 4; histologic grade n = 16; Lymph node metastasis: n = 5; HER2-status: n = 5, CK5/6: n = 2; molecular subtype: n = 6.

Variables	TFF1 negative (SI 0) n(%)	TFF1 positive (SI 1–9) n(%)	OR	CI 95%	P-value
Age					** < 0.001**
≥ 40 years (older)	49 (20.3)	192 (79.7)	1		
< 40 years (younger)	33 (42.3)	45 (57.7)	2.87	1.66–4.97	
Tumor diameter					0.392
≤ 20 mm	43 (23.9)	137 (76.1)	1		
> 20 mm	38 (28.1)	97 (71.9)	1.25	0.75–2.07	
Histologic grade					** < 0.001**
Low grade (grade 1/2)	33 (15.7)	177 (84.3)	1		
High grade (grade 3)	41 (44.1)	52 (55.9)	4.23	2.43–7.35	
Lymph node status					0.640
LN negative	41 (24.4)	127 (75.6)	1		
LN metastasis	39 (26.7)	107 (73.3)	1.13	0.68–1.88	
Estrogen receptor (ER) status					** < 0.001**
ER positive	22 (10.3)	192 (89.7)	1		
ER negative	60 (57.1)	45 (42.9)	11.64	6.47–20.92	
Progesterone Receptor (PR) status					** < 0.001**
PR positive	25 (11.6)	191 (88.4)	1		
PR negative	57 (55.3)	46 (44.7)	9.47	5.36–16.74	
HER2 status					0.103
HER2 negative	64 (24.3)	199 (75.7)	1		
HER2 positive	18 (35.3)	33 (64.7)	1.67	0.895–3.22	
CK 5/6 status					** < 0.001**
CK 5/6 negative	45 (17.0)	219 (83.0)	1		
CK 5/6 positive	37 (69.8)	16 (30.2)	11.25	5.77–21.96	
Molecular subtype (IHC)*					** < 0.001**
Luminal A	14 (11.5)	108 (88.5)			
Luminal B/HER2-	10 (11.0)	81 (89.0)			
Luminal B/HER2 +	5 (20.0)	20 (80.0)			
HER2 + non-luminal	13 (50.0)	13 (50.0)			
Triple negative	40 (81.6)	9 (18.4)			

*According to the 2013 St. Gallen Guidelines 2013^65^. Significant values are in bold.

In the subset of the METABRIC cohort within a corresponding age range (below 50 years, n = 204), low TFF1 mRNA expression (defined by the lower quartile cut-point) was similarly associated with younger age (< 40 years), higher histologic grade, and ER negative tumors, and was additionally associated with LN metastasis (OR ≥ 2.75, all P ≤ 0.004, Table 2, Supplementary Figures S1a-d). No association was observed between TFF1 and tumor size in any of the cohorts.

**Table 2:** Association between TFF1 expression (mRNA) and selected tumor characteristics in breast cancer (n = 204; METABRIC cohort, patients < 50). n = number of patients; LN: lymph node; p-values by Pearson’s chi-square test.

Variables	TFF1 low (Q1)	TFF1 high (Q 2–4)	OR	(CI 95%)	P-value
	n(%)	n(%)			
Age					**0.004**
≥ 40 years (older)	46 (30.5%)	105 (69.5)	1		
< 40 years (younger)	28 (52.8)	25 (47.2)	2.75	(1.35–4.85)	
Tumor diameter					0.136
≤ 20 mm	29 (30.9)	65 (69.1)	1		
> 20 mm	45 (40.9)	65 (59.1)	1.55	(0.87–2.77)	
Histologic grade					** < 0.001**
Low grade (grade 1/2)	6 (7.7)	72 (92.3)	1		
High grade (grade 3)	68 (54.0)	58 (46.0)	14.07	(5.70–34.73)	
Lymph node status					** < 0.001**
LN negative	21 (20.8)	80 (79.2)	1		
LN metastasis	53 (51.5)	50 (48.5)	4.04	(2.18–7.48)	
Estrogen receptor (ER) status					** < 0.001**
ER positive	8 (6.5)	115 (93.5)	1		
ER negative	66 (81.5)	15 (18.5)	63.25	(25.46–157.12)	
Molecular subtype (PAM50)*					** < 0.001**
Luminal A	4 (4.3)	88 (95.7)			
Luminal B	2 (6.1)	31 (93.9)			
HER2 type	16 (64)	9 (36.0)			
Basal-like	52 (96.3)	2 (3.7)			

*According to the PAM50 classification^34^. Significant values are in bold.

In survival analyses, negative TFF1 protein expression was associated with poorer survival in the Bergen Young BC Cohort (P = 0.05, Fig. 1d). Likewise, low TFF1 mRNA expression was significantly associated with reduced survival in the METABRIC cohort (P < 0.001, Fig. 1e). In multivariate Cox’ regression analyses, TFF1 mRNA expression remained independently associated with BC specific survival, when adjusting for histologic grade, tumor size and LN status (Supplementary Table 1).

When ER status (IHC) was included in the model (METABRIC cohort), dichotomized TFF1 mRNA expression was no longer independently associated with survival, but TFF1 as a continuous variable continued to be associated with survival when ER was included as a variable (HR 1.2 (95% CI 1.0–1.3), P = 0.019, Supplementary Table 2). A significant interaction between TFF1 mRNA and ER was observed (P = 0.002). In the Bergen Young BC Cohort however, TFF1 protein expression did not show independent prognostic significance in multivariate analyses (Supplementary Table 3).

**Fig. 2:**
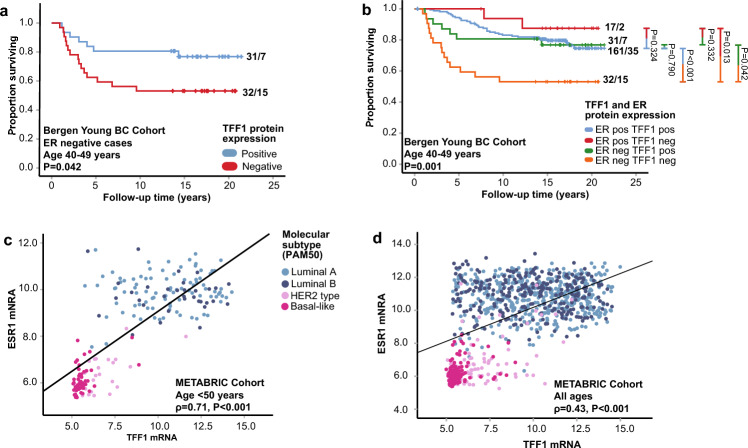
Association between TFF1 and ER expression and survival. (**a**) Association between TFF1 and survival in patients aged 40–49 years with ER negative tumors. (**b**) Association between ER and TFF1 status and survival in patients aged 40–49 years in the Bergen Young BC Cohort. (**c**, **d**) Correlation between TFF1 and ESR1 in patients < 50 years and in patients of all ages in the METABRIC cohort.

Given the association between low TFF1 mRNA expression and younger age, we stratified the survival analyses by age groups. In the Bergen Young BC Cohort, negative TFF1 protein expression was associated with shorter survival in patients aged 40–49 years (P = 0.029, Fig. 1f), but not for those younger than 40 years (P = 0.93, Fig. 1g). These findings were validated at the mRNA level (METABRIC cohort, age 40–49, P = 0.004, Supplementary Figure S1e; and age < 40, P = 0.097, Supplementary Figure S1f.). Furthermore, within the 40–49 age group, patients with tumors negative for both ER and TFF1 had significantly poorer survival compared to others (Bergen Young BC Cohort; P = 0.042, Fig. 2a). In the METABRIC cohort, patients with combined ESR1 mRNA-low and TFF1 mRNA-low tumors had the most reduced disease-specific survival probability, both among all patients under 50 years and within the age group 40–49 (All ages: P < 0.001, Supplementary Figure S1g; Age 40–49: P = 0.011, Supplementary Figure S1h). Interestingly, patients with combined ER positive/TFF1 negative/low tumors showed the most favorable survival outcomes within the 40–49 age group in both the Bergen Young BC and METABRIC cohorts (P ≤ 0.011, Fig. 2b, Supplementary Figure S1h).

### Gene regulation in TFF1 mRNA-low tumors

To get a deeper understanding of TFF1 expression patterns, we performed SAM analyses on TFF1 mRNA data. The estrogen receptor α gene (*ESR1)*, and some of its target genes, including Trefoil Factor 3 (*TFF3*), Anterior Gradient 3 (*AGR3*), Forkhead box A1 (*FOXA1*) and *GATA3*, were among the top-ranked downregulated genes in TFF1-low cases (Supplementary Table 4–5). Expression of these ER-related genes positively correlated with TFF1 expression both in the METABRIC cohort (Supplementary Figures S2a-d, ρ ≥ 0.68, P < 0.001) and the CCLE data set (Supplementary Figures S3a-d, ρ ≥ 0.63, P < 0.001).

Comparison of ESR1 and TFF1 mRNA expression revealed that tumors with concurrently low ESR1 and TFF1 expression were enriched in the basal-like subtype, both among patients below 50 years and across all age groups (METABRIC cohort; age < 50: ρ = 0.71, P < 0.001, Fig. 2c; all ages: ρ = 0.43, P < 0.001, Fig. 2d), as well as in BC cell lines from the CCLE data set (ρ = 0.68, P < 0.001, Supplementary Figure S3e). Tumors and cell lines with low ESR1 expression consistently exhibited low TFF1 expression, whereas in ESR1-high cases, the TFF1 expression ranged from low to high. This pattern suggests a context-dependent interplay between TFF1 and ER signaling.

### Low TFF1 expression associates with features of stemness and plasticity, and a basal-like phenotype

**Fig. 3:**
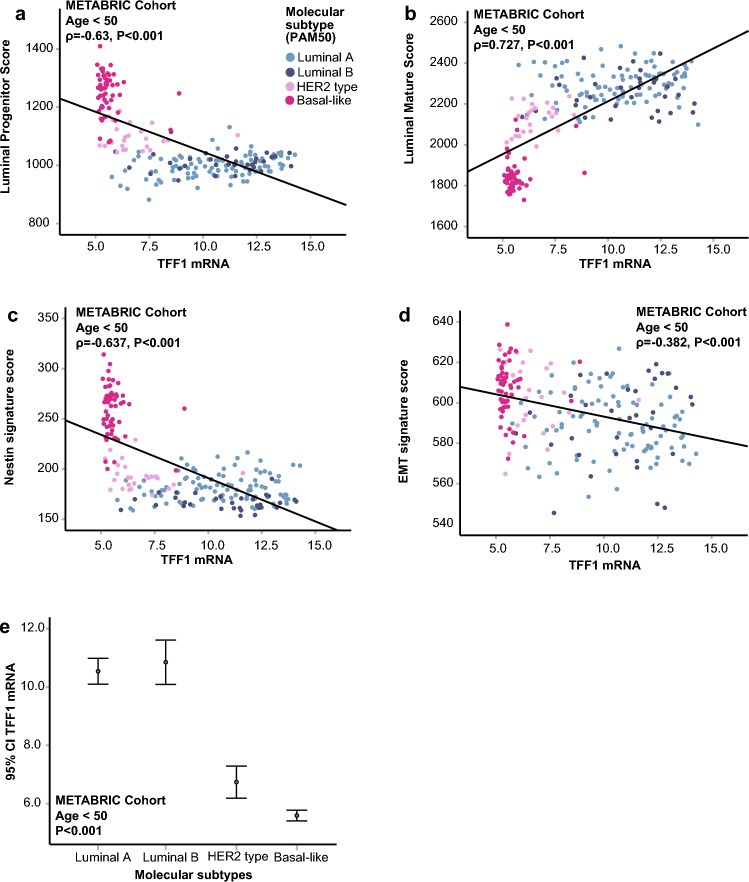
Gene expression signatures and molecular subtypes and association with TFF1 expression in the METABRIC cohort. (**a–d**) Association between TFF1 mRNA and luminal progenitor and maturity scores and stemness scores. (**e**) Association between TFF1 expression and molecular subtypes.

To explore the biological features associated with low TFF1 expression, we conducted gene set enrichment analyses (GSEA), which revealed significant enrichment of gene sets reflecting stemness and metastasis in TFF1-low tumors (GSEA MSigDB/C2 module, curated gene sets; Supplementary Table 6). These tumors also showed molecular signatures indicative of less differentiated state, including high luminal progenitor (ρ = −0.63, P < 0.001, Fig. 3a) and low luminal mature scores^31^ (ρ = 0.73, P < 0.001, Fig. 3b), as well as elevated Nestin^32^ (P < 0.001, ρ = −0.64, Fig. 3c) and EMT scores^33^ (ρ = −0.38, P < 0.001, Fig. 3d). In parallel, low TFF1 mRNA expression was correlated with a high Nestin score in cancer cell lines, further supporting an association with stem-like properties (CCLE; ρ = −0.83, P < 0.001, Supplementary Figure S3f.).

Verifying these findings, we demonstrated significant associations between negative TFF1 protein expression and the triple negative subtype (P < 0.001) and CK5/6 positivity (OR 11.25, (95% CI 5.77–21.96), P < 0.001) in PTs from the Bergen Young BC Cohort (both P < 0.001, Table 1). Consistently, low TFF1 mRNA expression was associated with a basal-like phenotype in the METABRIC cohort (P < 0.001, Fig. 3e, Table 2).

Multivariate logistic regression analyses based on gene expression data confirmed that both TFF1 mRNA and ER protein expression were independent predictors of the basal-like subtype (both P ≤ 0.003), with low TFF1 mRNA emerging as the stronger predictor (METABRIC cohort; OR 41.4 (95% CI 8.3–206.4), P < 0.001,Supplementary Table 7), compared to ER-negative (OR 9.0 (95% CI 2.1–38.4), P = 0.003, Supplementary Table 7) respectively. The interaction between ER and TFF1 in these analyses was not statistically significant (P = 0.93). Also among patients below 40 years of age, when both TFF1 and ER were included in the multivariate logistic regression model, TFF1 remained a significant predictor of the basal-like subtype, while ER did not (TFF1 mRNA: OR 49.28 (95% CI 3.91–621.58), P = 0.003; ER: OR 4.1 (95% CI 0.23–72.22), P = 0.34). Significantly, multivariate logistic regression evaluating the predictive value of TFF1 alongside other basal-like subtype markers, showed TFF1 mRNA expression to be a stronger predictor of the basal-like subtype (dichotomized TFF1: OR 104.8 (95% CI 13.2–830.7), P < 0.001) than CK5 (OR 3.8 (95% CI 1.7–9.2), P = 0.001), EGFR (not significant) and P-cadherin (not significant) in patients under 50 years (Supplementary Table 8). Additionally, TFF1 and ER protein expression both predicted CK5/6 expression, a surrogate marker for the basal-like subtype, in the Bergen Young BC Cohort (TFF1: OR 5.4 (95% CI 2.4.11.3), P < 0.001; ER: OR 5.3 (95% CI 2.4–11.5), Supplementary Table 9).

In the full METABRIC cohort (n = 939), ER expression was a stronger predictor of the basal-like subtype than TFF1 mRNA, with ER showing a hazard ratio (HR) of 28.0 (95% CI 13.7–57.2) compared to TFF1’s HR of 15.9 (95% CI 7.3–34.6). This is in line with how the basal-like subtype is defined^34,35^, further underscoring biological differences associated with age (Supplementary Table 10).

Building on our understanding of the role of TFF1 mRNA expression independent of ER status, we compared differentially expressed genes in TFF1-low and -high cases, and ER-negative and positive tumors in patients aged < 50 years in the METABRIC cohort. In tumors with concurrent ER negative and low TFF1 mRNA expression, 27 genes were uniquely up-regulated, and 29 genes were uniquely down-regulated (Fold change > 2.0, Supplementary Table 11–12). Among up-regulated genes were genes encoding intermediate filament proteins, such as *KRT5* and *KRT17* (Fold change ≥ 2.0, Supplementary Table 11). These keratins are key structural components of the cytoskeleton, and keratins (CKs) have been implicated in metastasis, showing both up- and downregulation, depending on context^36^. Specifically, CK5 and CK17 characterize the basal-like BC subtype^37^, reinforcing the notion that TFF1 may be a stronger predictor of this subtype than ER. In support of this, analyses of the Bergen Young BC Cohort revealed a significant association between negative TFF1 expression and positive CK5/6 status (69.8% (37/53), OR 11.25, (95% CI 5.77–21.96), P < 0.001, Table 1).

### TFF1-low tumors associate with proliferation and immune evasion

**Fig. 4:**
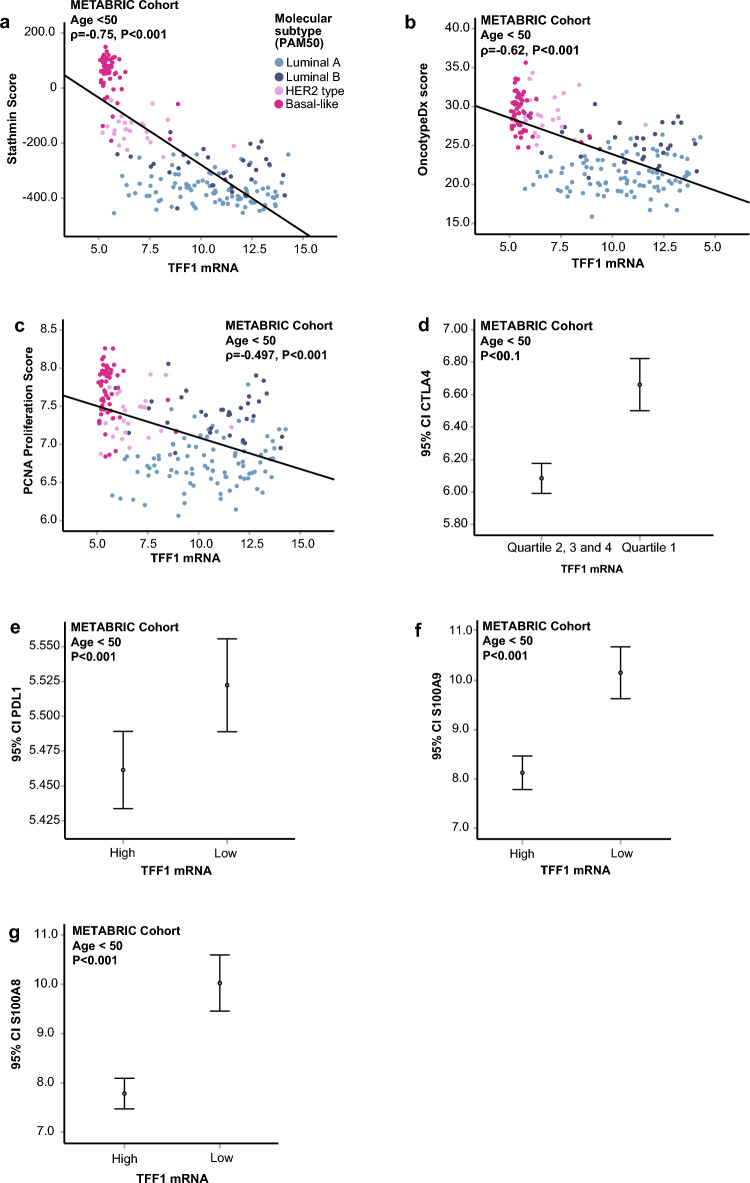
Associations between TFF1 mRNA and proliferation scores and immune markers in the METABRIC cohort. (**a**-**c**) Associations between TFF1 and proliferation scores Stathmin, OncotypeDx and PCNA. (**d**-**g**) Association between TFF1 and immune markers.

GSEA revealed strong enrichment of proliferation-related gene sets in TFF1 mRNA-low tumors (GSEA MSigDB modules; C2 – curated gene sets, C5 – biological processes, and H – hallmarks; FDR(%) range 0.14–4.46, Supplementary Tables 6, 13, 14), particularly processes regulating various phases of the cell cycle. In line with this, TFF1 mRNA expression correlated negatively with several proliferation scores, including the Stathmin score^38^ (ρ = −0.75, P < 0.001, Fig. 4a), Oncotype Dx^39^ (ρ = −0.62, P < 0.001, Fig. 4b), and a PCNA score^40^ (ρ = −0.5, P < 0.001, Fig. 4c). Analyses of BC cell line data also demonstrated inverse correlations between TFF1 mRNA expression and both the Stathmin (ρ = −0.83, P < 0.001, Supplementary Figure S3g) and PCNA scores (ρ = −0.43, P = 0.002, Supplementary Figure S3h).

Gene sets related to both innate and adaptive immune responses were significantly enriched in TFF1-low tumors (GSEA MsigDB modules C5 and H; FDR (%) range 0.37–4.46, Supplementary Tables 13–14). Additional analysis revealed that low TFF1 mRNA expression was significantly associated with elevated expression of key immune checkpoints and inflammatory markers, including CTLA4, PD-L1, S100A8 and S100A9 (All P < 0.001, Fig. 4 d-g), suggesting the presence of an immune evasive tumor environment.

### TFF1 expression in lymph node metastases

To determine whether TFF1 expression changes during cancer progression – from primary lesion to metastatic sites – we assessed TFF1 protein expression in paired samples of PT and axillary LN metastases from the Bergen Young BC Cohort. Among the 319 patients included in the cohort, 52% (166) had axillary LN metastases at diagnosis. For the analyses, we included patients whose first LN metastasis occurred ipsilaterally (n = 145; Supplementary Figure S4). Immunohistochemical evaluation of TFF1 protein expression was successfully performed in matched PT and LN metastases for 90.3% (131/145) of these cases.

Among the 145 LN metastases analyzed, 83.4% (121/145) exhibited positive TFF1 expression, while 16.6% (24/145) showed no detectable expression. A strong concordance was observed between positive TFF1 protein expression in the PT and their corresponding LN metastases (95.8%, P < 0.001, Supplementary Table 15). Negative TFF1 protein expression in the LN metastases was significantly associated with high histologic grade, as well as ER and PR negativity in the PT (OR ≥ 4.57, all P ≤ 0.001, Table 3). Moreover, negative TFF1 protein expression in the LN metastases was associated with the triple negative subtype and CK5/6 positivity in the PT (CK5/6: OR 16.25 (95% CI 5.71–46.23) P < 0.001, Table 3). The level of TFF1 protein expression in LN metastases was not associated with patient age, tumor diameter, or HER2 status of the primary tumor.

**Table 3:** Association between TFF1 expression (IHC) in lymph node metastases and selected characteristics in breast cancer (n = 145; Bergen Young BC Cohort). Cut-point for positive expression: 5 cells (n = 145). n = number of patients, LN = lymph node; p-values by Pearson’s chi-square test. Missing information: Histologic grade: n = 7; TFF1 expression in primary tumors: n = 14; CK5/6 status: n = 6; HER2 status: n = 9; molecular subtype: n = 9.

Variables	TFF1 negative (SI 0) in LN metastases n(%)	TFF1 positive (SI 1–9) in LN metastases n(%)	OR	CI 95%	P-value
Age					0.071
≥ 40 years (older)	13 (12.9)	88 (87.1)	1		
< 40 years (younger)	11 (25.0)	33 (75.0)	2.26	(0.92–5.53)	
Tumor diameter in primary tumor					0.847
≤ 20 mm	10 (15.9)	53 (84.1)	1		
> 20 mm	14 (17.1)	68 (82.9)	1.09	(0.45–2.65)	
Histologic grade in primary tumor					**0.001**
Low grade (grade 1 + 2)	7 (8.1)	79 (91.9)	1		
High grade (grade 3)	15 (28.8)	37 (71.2)	4.57	1.72–12.17	
TFF1 expression in primary tumor					** < 0.001**
TFF1 high (SI 1–9)	4 (4.2)	92 (95.8)	1		
TFF1 (low (SI 0)	17 (48.6)	18 (51.4)	21.72	(6.54–72.17)	
Estrogen receptor (ER) status in primary tumor					** < 0.001**
ER positive	3 (3.2)	90 (96.8)	1		
ER negative	21 (40.4)	31 (59.6)	20.32	(5.67–72.85)	
Progesterone Receptor (PR) status in primary tumor					** < 0.001**
PR positive	3 (3.2)	91 (96.8)	1		
PR negative	21 (41.2)	30 (58.8)	21.23	(5.92–76.23)	
CK 5/6 status in primary tumor					** < 0.001**
CK 5/6 negative	8 (7.1)	104 (92.9)	1		
CK 5/6 positive	15 (55.6)	12 (44.4)	16.25	(5.71–46.23)	
HER2 status in primary tumor					0.613
HER2 negative	19 (17.8)	88 (82.2)	1		
HER2 positive	4 (13.8)	25 (86.2)	0.74	(0.23–2.38)	
Molecular subtype					** < 0.001**
Luminal A	1 (2.2)	44 (97.8)			
Luminal B/HER2-	2 (5.0)	38 (95.0)			
Luminal B/HER2 +	0 (0.0)	11 (100.0)			
HER2 + non-luminal	4 (22.2)	14 (77.8)			
Triple negative	16 (72.7)	6 (27.3)			

Significant values are in bold.

**Fig. 5:**
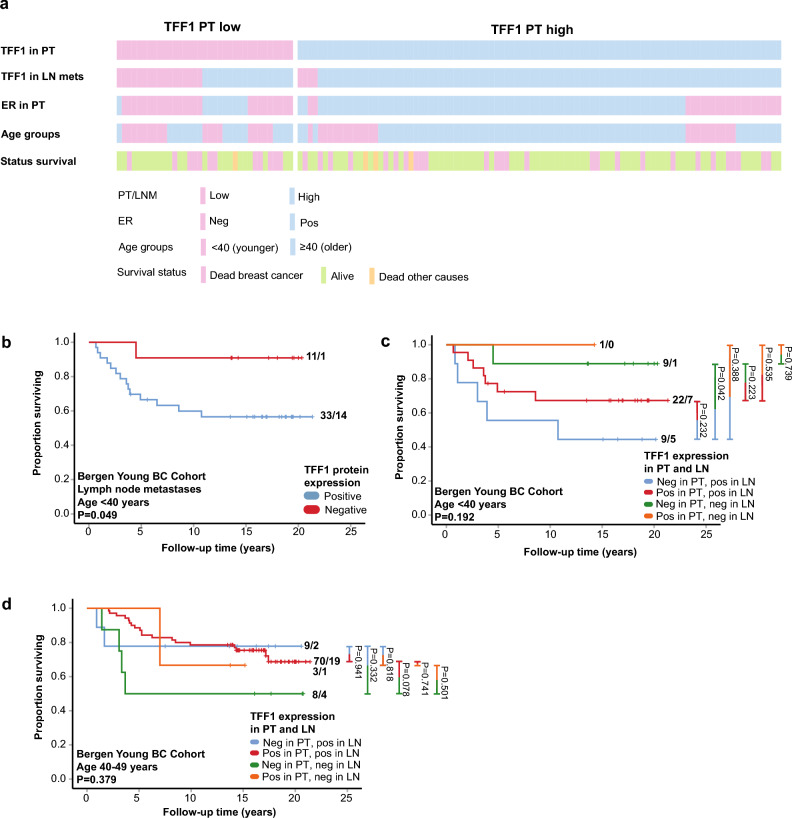
Overview of TFF1 IHC expression and association with key clinical variables. Association between age, ER status in PT, TFF1 status in both PT and LN metastases, survival and histological subtype in the Bergen Young BC Cohort. See colors in key for variable details. Vertical lines correspond to data for one patient with variables aligned horizontally. (**b**) Survival of patients under 40 years based on TFF1 expression in LN metastases. (**c**, **d**) Survival of patients under 40 years and 40–49 years based on TFF1 expression in PT and LN metastases.

Discordant TFF1 protein expression between the PT and the matched LN metastasis was observed in 16.8% of patients (22/131, Supplementary Table 15; Fig. 5a). Among TFF1 negative PTs, 51.4% (18/35) showed TFF1 protein expression in their corresponding LN metastases (Supplementary Table 15), whereas only 4.2% (4/96) of TFF1 positive PTs had TFF1 negative LN metastases (Supplementary Table 15). Concurrent TFF1 negative protein expression in PTs and the matched LN metastases was significantly associated with younger patient age, ER and PR negativity, CK5/6 positivity, and the TNBC subtype (P ≤ 0.029, Supplementary Table 16). There were few cases in the discordant groups, making the statistical power too weak to conclude on the association with clinico-pathologic variables (Supplementary Table 16).

Similarly to TFF1 negative tumors, 59,6% (31/52) of the ER negative PT showed positive TFF1 expression in the LN metastases (OR 20.3 (CI 95% 5.7–72.9), P < 0.001). Among these, 67.9% (19/28) also presented positive TFF1 expression in the primary tumors. Notably, among cases with concurrent ER negative and TFF1 negative PT, 36% (9/25) of patients demonstrated positive TFF1 protein expression in the LN metastases (Fig. 5a).

Among patients younger than 40 years, positive TFF1 protein expression in LN metastases was associated with reduced survival (P = 0.049, Fig. 5b). This association was not observed in patients aged 40–49 years (P = 0.119, Supplementary Figure S5a) or in the broader group of patients under 50 years (P = 0.801, Supplementary Figure S5b).

When stratifying patients by concordance of TFF1 expression, individuals under 40 years with TFF1 negative PT and TFF1 positive LN metastases had reduced survival compared to those with TFF1 negative expression in both the PT and LN metastases (P = 0.042, Fig. 5c). In contrast, among patients aged 40–49 years, there was a trend toward reduced survival in those with negative TFF1 expression in both the PT and LN metastases, compared to those with TFF1 positivity in both sites (P = 0.078, Fig. 5d). These findings underscore age-dependent differences in the prognostic relevance of TFF1 expression during tumor progression.

## Discussion

Low TFF1 expression has previously been associated with more aggressive tumor features^8,41^, but its role in young BC patients has not been explored. Younger patients are known to present with a more aggressive tumor phenotype, characterized by more frequent loss of ER and PR expression^4,42^ and increased expression of HER2 and EGFR, compared to older patients^43^. In this study, we demonstrate that low TFF1 protein and gene expression is associated with aggressive clinico-pathologic tumor features and age. In agreement with these observations, reduced survival was observed among patients with TFF1-low tumors, both across all patients below 50 years and particularly within the subgroup of patients aged 40–49 years.

TFF1 is an estrogen-inducible protein, where ER binding to the TFF1 promoter induces transcription. In line with this, we observed a strong correlation between ER and TFF1 positivity both at the protein and mRNA levels in PTs (Bergen Young BC Cohort: 89.7%, METABRIC: 93.5%). However, some BC patients with ER negative tumors exhibited positive TFF1 expression, suggesting that these two factors may be regulated both cooperatively and independently. Previous studies have shown that TFF1 also can be regulated by other factors, like EGF, the HRAS oncoprotein and the c-jun protein^44^, which may explain TFF1 expression in the absence of ER. Notably, combined ER and TFF1 negativity in PT was associated with reduced survival only in the 40–49 age group in the Bergen Young BC Cohort, indicating a possible age-dependent aggressive tumor phenotype. In contrast, earlier studies of TFF1 expression in BC patients, where analyses were stratified by menopausal status or other age cut-offs, have generally not shown a correlation between TFF1 status and age^8,45–50^.

In gastric and pancreatic tissue, where TFF1 has been most extensively studied, TFF1 exhibits tumor-suppressive properties^51,52^, and its loss is frequently observed in gastric cancer^53^. A recent study of pancreatic cancer further demonstrated that TFF1 suppresses stemness^54^, suggesting that low TFF1 may serve as a marker for stem-like features. In our study, we found that low TFF1 mRNA expression was a stronger predictor of the basal-like subtype than ER expression and other established basal-like BC markers in patients under 50 years of age. However, when older patients (> 50 years) were included, ER became the better predictor, indicating an age-dependent difference in TFF1 expression and function, and that TFF1 might be a potential prognostic marker for patients in this age group.

Genes regulating proliferation, stemness, and immune evasion were significantly upregulated in TFF1-low tumors, further supporting TFF1-low as a marker for more aggressive disease. Additionally, genes characteristic of the basal-like subtype, including keratin coding genes^55,56^, were more strongly upregulated in TFF1-low tumors compared to ER negative tumors. Previous studies have also shown that TFF1-low expression is associated with triple negative BC and poorer outcome compared to TFF1-high tumors^8,57^, and low TFF1 expression in node-negative BC patients is linked to an increased risk of relapse^58^.

Given the critical role of metastases in BC progression, we looked at patients with matched LN metastases and found a high concordance rate (95.8%) of positive TFF1 expression between PTs and corresponding LN metastases. This finding aligns with the results from Nichols *et.al.,* who reported a strong correlation between positive TFF1 in PTs and LN metastases, concluding that the TFF1 (pS2) status of LN metastases can be predicted based on the TFF1 status of the PT^19^.

Interestingly, TFF1 positivity in LN metastases arising from PTs that were ER and TFF1 negative, were associated with poorer survival than TFF1 negativity, but only in patients under 40 years of age. Despite the small sample size, which limits statistical power of certain conclusions, and lack of detailed histopathologic variables for LN metastases, it is tempting to speculate that the TFF1 re-expression in metastases may occur independently of ER, potentially driven by epigenetic mechanisms such DNA methylation. However, these findings should be interpreted with caution. Further studies are required to verify the clinical significance of this finding, elucidating the underlying regulatory mechanisms, and consider age-related influence of TFF1 expression during cancer progress.

We recognize the limitations of our molecular analyses, including GSEA, stemness signatures, and immune-related markers, as these were conducted using external datasets (METABRIC and CCLE) rather than the Bergen Young BC Cohort. Consequently, protein-level findings from the Bergen Young BC Cohort can only be indirectly associated with mRNA-level biology, as the patient samples do not overlap. While this approach is common and supported by previous studies^59,60^, such limitations should be acknowledged when interpreting mechanistic claims.

In summary, we demonstrate that TFF1-low is associated with aggressive clinico-pathologic features and poorer outcome and serves as a strong predictor of the basal-like subtype in younger women with BC. The enrichment of gene programs related to proliferation, metastasis, stemness, and immune evasion in TFF1-low tumors further supports TFF1 as a marker of aggressive phenotypes in this population, highlighting a context- and age-dependent relationship between ER and TFF1. Additionally, reduced survival among patients under 40 years with discordant TFF1 expression between PTs and LN metastasis suggests that TFF1 may have prognostic value in this subgroup.

Our findings offer clinically relevant insights and serve as a foundation for hypothesis-driven research. Future studies should incorporate functional validation and larger independent cohorts to confirm and extend these observations. Our descriptive and correlative approaches are critical for hypothesis generation and for identifying biomarkers that may guide subsequent mechanistic and translational research.

## Materials and methods

### Patient cohorts

The study includes a population-based in-house cohort – The Bergen Young BC Cohort – comprising formalin-fixed, paraffin embedded (FFPE) primary invasive BC tissue samples (n = 355) from patients below 50 years of age in Hordaland County, Norway, diagnosed in the period 1996–2003. All aspects of this study were performed in accordance with ethical guidelines and regulations as stated in the Ethics section. Clinico-pathologic variables and further details about this cohort have previously been described by our group^42^. Information on the clinico-pathologic variables were obtained through the local pathology registry (Department of Pathology, Haukeland University Hospital, Bergen, Norway) and the Cancer Registry of Norway^61^. The follow-up information, acquired from the Norwegian Cause of Death Registry, was considered accurate and complete, and included information on follow-up time, status at last follow-up, and cause of death. The last date of follow-up was June 30, 2017. Median follow-up time of survivors was 202 months (range 162–257 months). In brief, immunohistochemical data of the biomarkers ER and PR were obtained from the routine pathology reports or by additional immunohistochemistry (IHC)^42^. The cut points for ER and PR positivity were kept at 10% staining, in accordance with clinical practice at the time of diagnosis. The established scoring system for DAKO Herceptest was used^62^. HER2 silver In Situ Hybridization (SISH) was performed on IHC 2 + cases (Ventana INFORM HER2 DNA probe staining). Cases were scored 0, 1 +, 2 + or 3 + based on intensity grade of membrane staining and percentage of tumor cells stained. Cases with 0 and 1 + scores were classified as negative, and 3 + as positive. HER2 SISH was performed on IHC 2 + cases (Ventana INFORM HER2 DNA probe staining). The 2 + cases were considered HER2 positive if the HER2/Chr17 ratio by SISH was ≥ 2.0^63,64^. Ki67 was assessed by IHC on tissue microarrays (TMA) or whole sections when the case was not available in the TMA. The molecular subtypes were originally classified using ER, PR, HER2 and Ki67 immunohistochemical markers, as recommended in the St. Gallen Guidelines 2013^42,65^.

166 of the patients (47.4%) in this cohort presented with one or more axillary metastases. Only patients with ipsilateral LN metastasis that occurred < 1 year after primary diagnosis were considered (n = 159). An additional 14 patients were excluded based on lack of sufficient tumor material or unavailable tissue samples, leaving 145 LN metastases available for analyses (Supplementary Figure S4).

### Immunohistochemistry protocol

TFF1 immunohistochemistry (IHC) was applied to 4–5 μm tissue microarray (TMA) slides from the PT in the Bergen Young BC Cohort. We used three cores (diameter 1.0 mm) of formalin-fixed paraffin-embedded (FFPE) primary BC tissue from each patient^42^. For LN metastases, whole sections were used for TFF1 staining. The slides were deparaffinized in xylene and rehydrated in 100%, 96% and 80% ethanol solutions. We performed epitope retrieval using microwave oven heating (6^th^ sense) in a solution target retrieval of pH6 (S1699) for 20 min. The slides were cooled off at room temperature, before applying a peroxidase block (S2023) to reduce background staining. The slides were then incubated with a 1:100 solution of a monoclonal rabbit anti-TFF1 antibody (ab92377) and diluent (Dako S3022) at room temperature for 30 min and thereafter incubated with a EnVision +/HRP anti-rabbit (K4003) secondary antibody for an additional 30 min. DAB chromogen (K3468) was used to visualize binding of the antibody. The slides were counterstained using hematoxylin (S3301) and then dehydrated by rinsing in ethanol (80%, 96% and 100%, respectively) and xylene. The same protocol was used for whole sections staining of PT and axillary LN metastases.

### Staining evaluation

There were 329 cases in the cohort with available tumor blocks. Further 10 cases were excluded due to insufficient tumor material for IHC staining, leaving 319 PT samples available for staining evaluation. The TFF1 immunohistochemical staining showed a heterogenous expression pattern. The staining was evaluated using a semi-quantitative, subjective staining system, where a score from 0–3 was given both for the intensity of the staining (0 = none, 1 = weak, 2 = moderate, 3 = strong) and the fraction of stained tumor cells (< 10% = 1, 10–50% = 2, > 50% = 3). A staining index (SI) was calculated as the product of the intensity and area of stained tumor cells (values 0–9). TFF1 expression was considered positive at SI 1–9, and negative at SI 0 for both PT and LN metastases, based on frequency distributions of the score indices and evaluating frequency of case-distribution of clinico-pathologic variables and survival patterns. Only expression of TFF1 in invasive breast carcinoma was considered in the analyses. For the axillary LN metastasis, we set a cut-point for positive staining at five positive tumor cells.

#### Gene expression resources and analyses

To explore the gene expression patterns related to TFF1 in BC, we applied a publicly available microarray mRNA gene expression dataset: The Molecular Taxonomy of Breast Cancer international Consortium METABRIC Discovery cohort (n = 939)^66^. This cohort consists of mRNA data from primary invasive BC and includes clinico-pathologic data, molecular subtypes, and long-term clinical follow-up (median follow-up time for survivors 122 months; range 1–270). Molecular subtypes were identified using the PAM50 classification^34^. Cases of the normal-like subtype were excluded from analyses. For validation, we analyzed BC cell line mRNA data from the Cancer Cell Line Encyclopedia (CCLE) (n = 47), with information on molecular subtypes^67^.

Cut point for TFF1 mRNA gene expression was determined after considering frequency distribution and survival patterns of quartiles in the full METABRIC cohort. Cut point for TFF1-low was defined as the lower quartile (Q1). To enable comparison of results from the Bergen Young BC Cohort and the METABRIC cohort, we applied an age cut point at < 50 years in the METABRIC cohort, including only these patients (n = 204) for analyses.

To explore genes differentially expressed between TFF1-high and -low cases, we performed Significance Analysis of Microarrays (SAM)^68^. Gene Set Enrichment Analysis (GSEA) with datasets from the Molecular Signature Database (MSigDB; https://www.gsea-msigdb.org/gsea/msigdb) were applied to study gene sets enriched in TFF1-low cases^69^. From the MSigDB we applied the gene sets reflecting biological processes (GO/BP, C5), curated gene sets (C2)  and hallmarks (H). Only gene sets with 10–500 genes were included in the GSEA analysis.

To validate our findings from the explorative gene expression analyses, we applied previously described gene expression signature scores reflecting luminal mature and progenitor features^31^; scores reflecting tumor proliferation (a Stathmin score^38^; OncotypeDx^39^; and a Proliferating Cell Nuclear Antigen (PCNA) score^40^), and scores reflecting Epithelial-Mesenchymal transition (EMT)^33^ and stemness features^32^.

### Ethics declaration

We confirm that all methods were carried out in accordance with ethical guidelines and regulations. The study was approved by the Western Regional Committee for Medical and Health Research Ethics, REC West (approval 2014/1984). Informed consent was waived by the Regional Ethics Committee. The national identification numbers of all patients were checked with the Registry of Withdrawal from Biological Research Consent by the Norwegian Institute of Public Health. None of the cases in our study were listed in the registry.

### Statistical analyses

We performed statistical analyses using SPSS Statistics for Windows, Version 26.0 (Armonk, Y: IBM Corp.). A two-sided P-value less than 0.05 was interpreted as statistically significant. We evaluated associations between categorical variables by Pearson’s chi-square test and included calculations on odds ratio (OR) and 95% confidence interval (CI). To compare distribution of continuous variables across categorical variables, we used Mann–Whitney U test and Kruskal–Wallis H test. McNemar’s test was used to compare nominal paired data. Error-bars were presented with 95% CI of the mean. Bivariate continuous variables were compared using Spearman’s rank correlation test. In survival analyses, we used BC specific survival as endpoint in both the Bergen Young BC Cohort and the METABRIC cohort. Survival analyses were performed using the Kaplan–Meier method, assessing statistical significance by the log-rank test. Multivariate survival analyses were conducted using the Cox proportional hazards regression model, applying the enter method for variable inclusion. Interactions between variables was tested by using interaction terms in the regression model (a x b). Multiple logistic regression analyses were performed using the enter method to predict basal-like subtype in the cohorts, with p-values derived from the Wald test.

## Supplementary Information

Supplementary Information 1.

Supplementary Information 2.

## Data Availability

The public gene expression datasets (METABRIC cohorts) are available at https://ega-archive.org/studies/EGAS00000000083. The datasets generated during and/or analysed during the current study are not publicly available due to reasons of sensitivity. However, upon reasonable request, interested researchers may contact the corresponding author to inquire about access. Request for non-commercial use will be considered and will require full ethics review.
