# Supplementary material for: Low tumoral Trefoil Factor 1 expression relates to aggressive tumor features and poor survival in young women with breast cancer
Source: Sci Rep. 2026 Jan 17;16:5612. doi: 10.1038/s41598-026-36341-4 (PMC12891483; doi:10.1038/s41598-026-36341-4)
Supplement: Supplementary file 1 — Supplementary Information 1. [file 41598_2026_36341_MOESM1_ESM.pdf]

Supplementary Figure S1

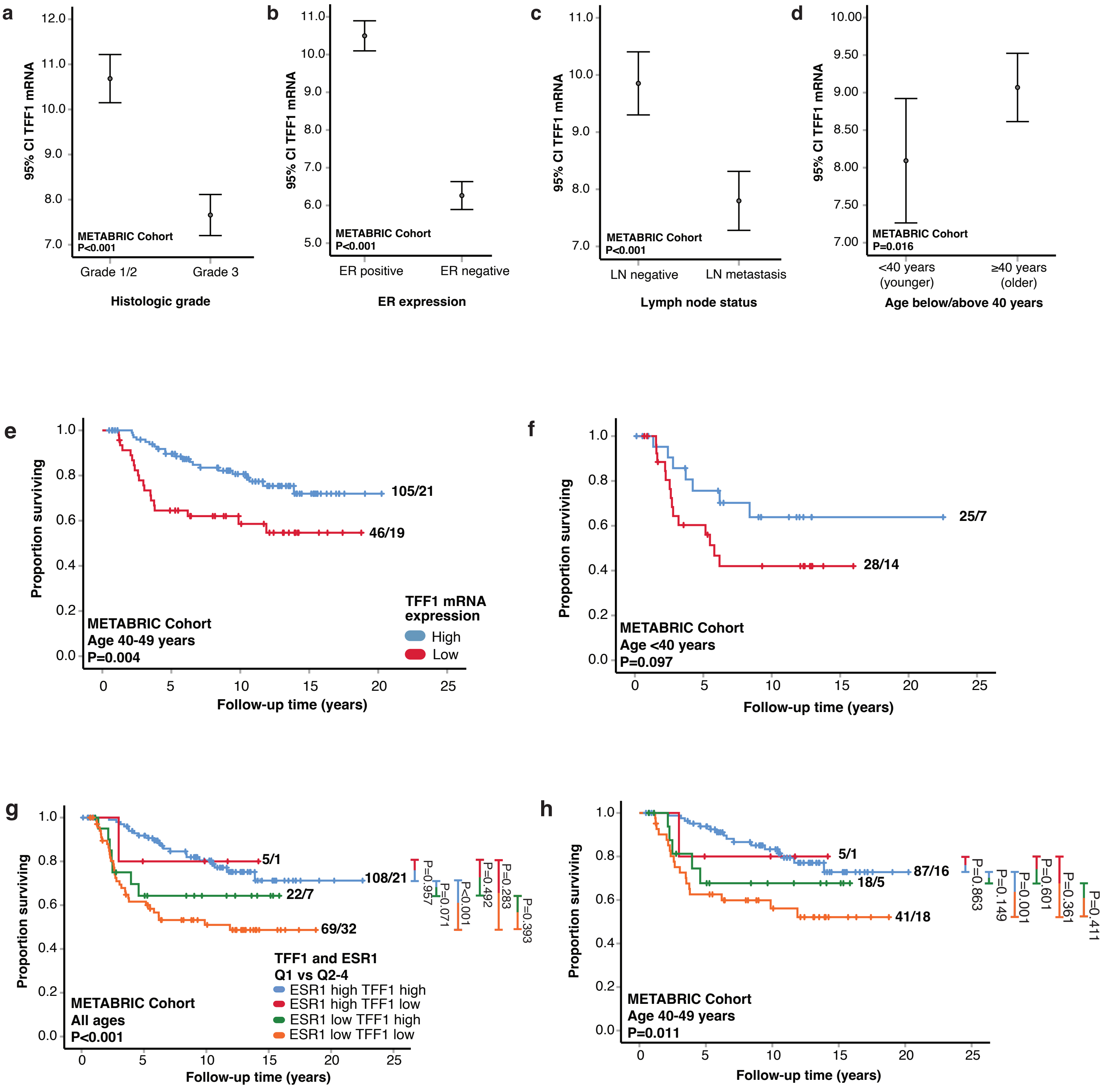

Supplementary Figure S1. TFF1 mRNA expression in relation to key variables and survival by age in the METABRIC cohort (n=204). (a-d) Association between TFF1 and the clinico-pathologic variables histologic grade, ER status, LN metastasis status, age in the METABRIC cohort. (e-f) Association between TFF1 and survival in patients aged 40-49 years and <40 years in the METABRIC cohort. (g-h) Association between TFF1 and ESR1 expression and survival in all patients <50 years and in patients aged 40-49 years

Supplementary Figure S2

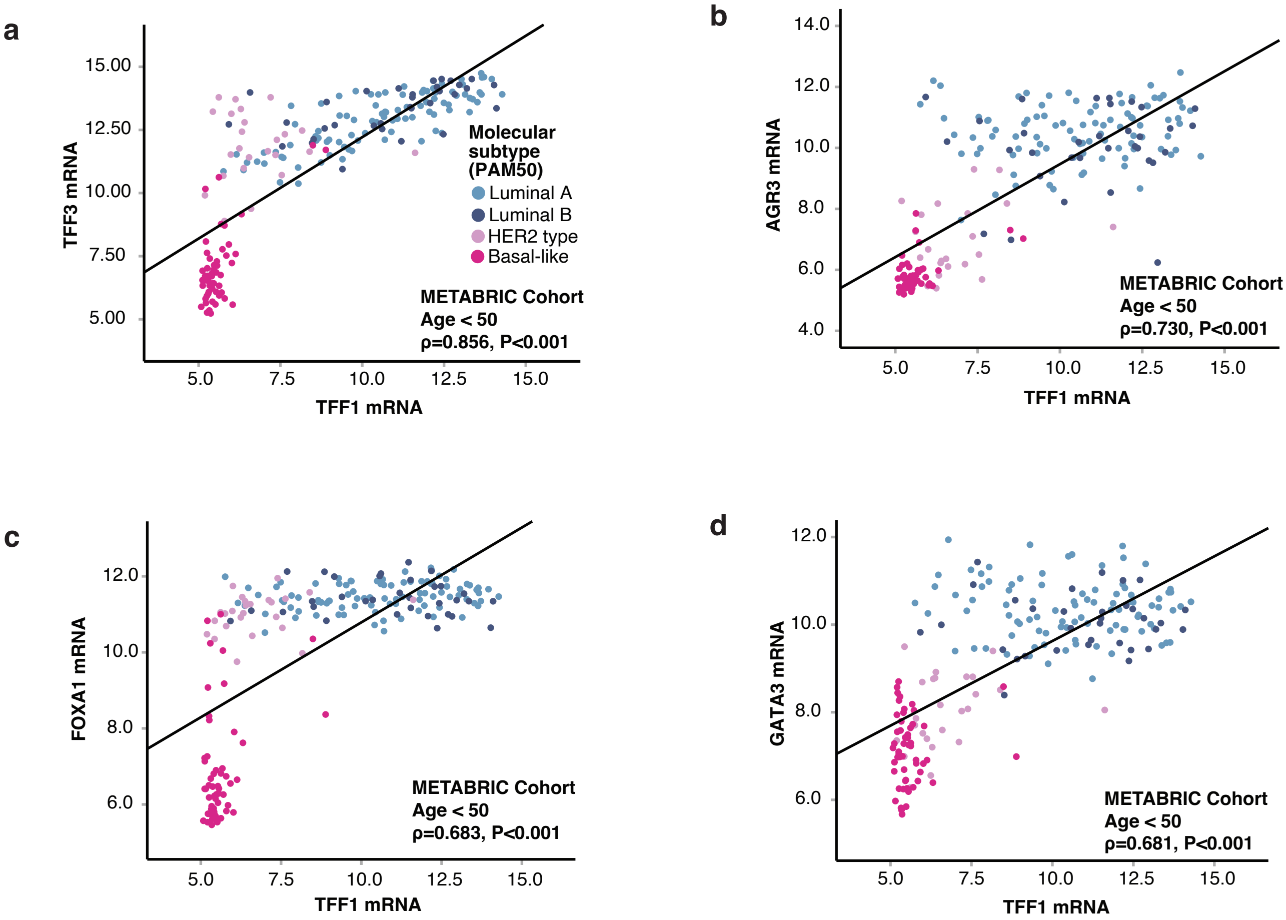

Supplementary Figure S2. Correlation between TFF1 and TFF3, AGR3, FOXA1 and GATA3 in the METABRIC cohort (n=204).

Supplementary Figure S3

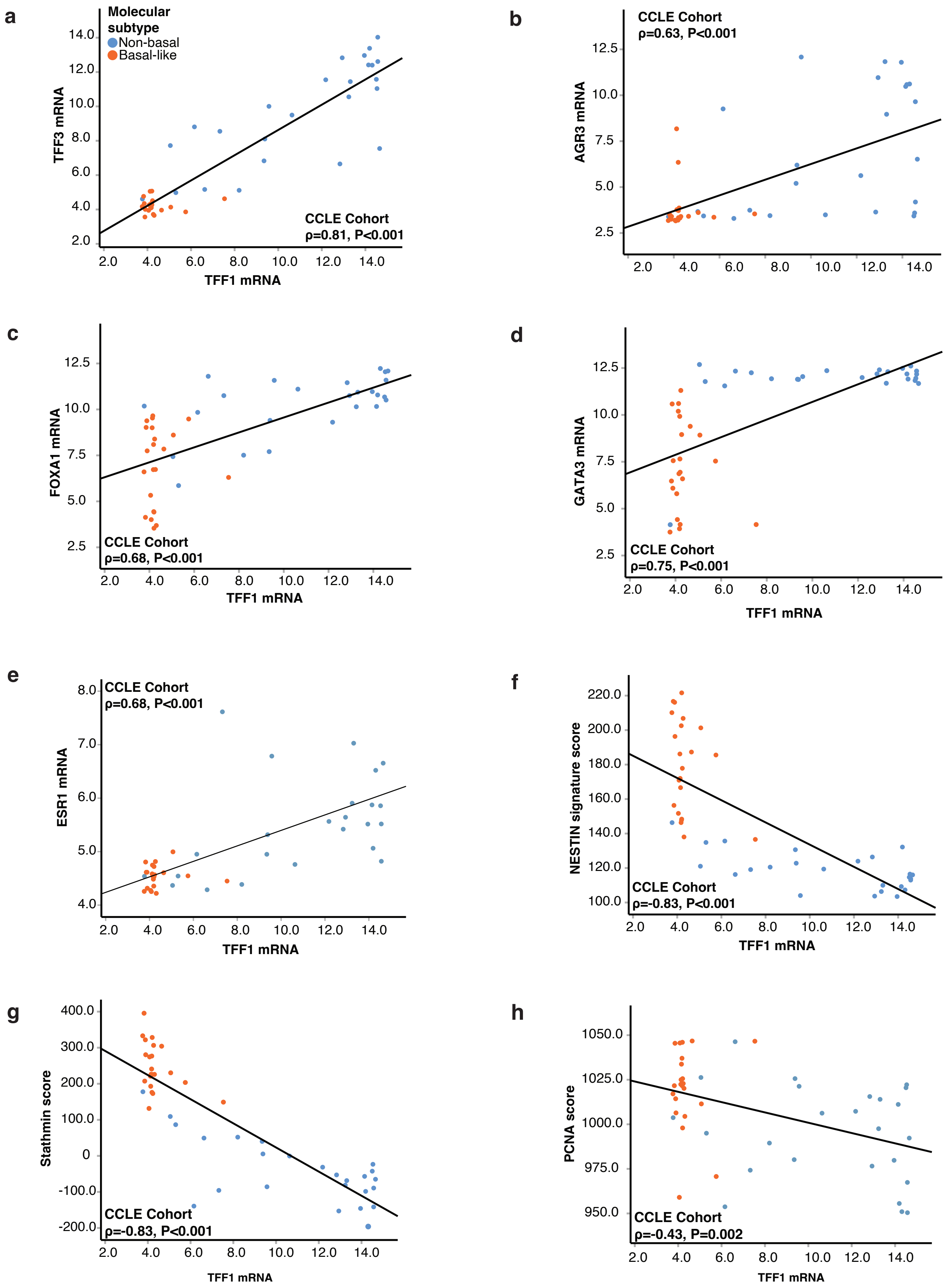

Supplementary Figure S3. Correlation between TFF1 and other genes and proliferation scores in the CCLE cohort (n=47). (a-e) Correlation between TFF1 and TFF3, AGR3, FOXA1, GATA3 and ESR1. (f) Association between TFF1 and stemness-score Nestin Signature score. (g-h) Association between TFF1 and proliferation scores Stathmin and PCNA.

Supplementary Figure S4

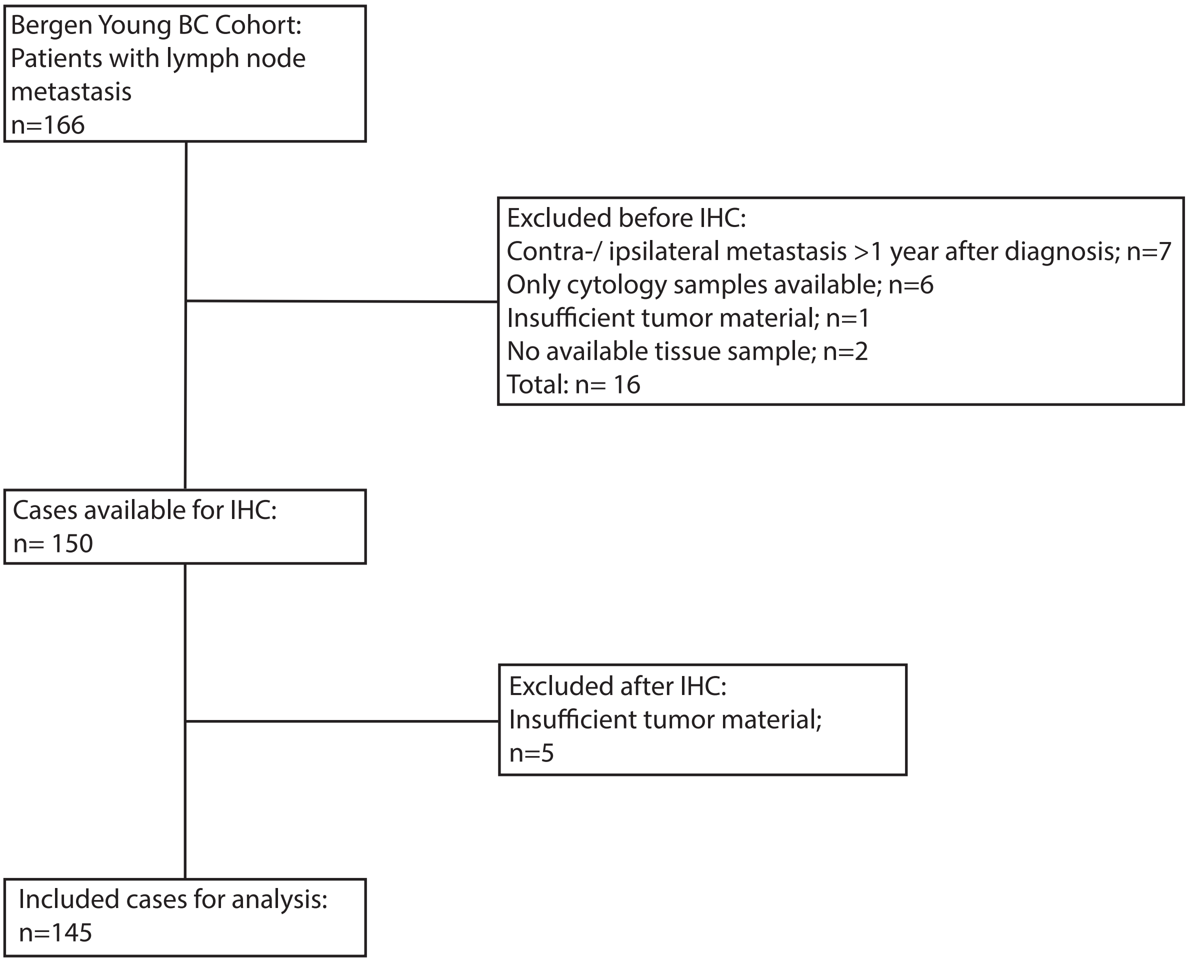

Supplementary Figure S4. Flow-chart of cases selected from the Bergen Young Cohort for analysis. A total of 166 patients had LN metastases and n=21 cases were excluded in total, leaving n=145 cases available for final analysis of the Bergen Young BC Cohort.

Supplementary Figure S5

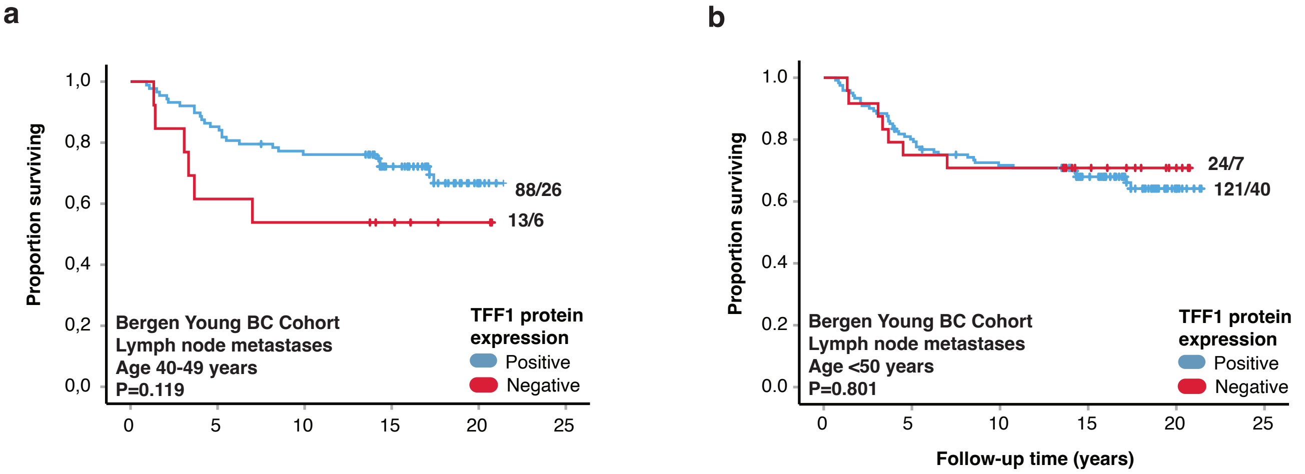

Supplementary Figure S5. (a-b) Survival of patients aged 40-49 and under 50 years of age based on TFF1 expression in LN metastases in the Bergen Young BC Cohort (n=145).
